# Homogenous overexpression of the extracellular matrix protein Netrin-1 in a hollow fiber bioreactor

**DOI:** 10.1007/s00253-021-11438-0

**Published:** 2021-08-03

**Authors:** Aniel Moya-Torres, Monika Gupta, Fabian Heide, Natalie Krahn, Scott Legare, Denise Nikodemus, Thomas Imhof, Markus Meier, Manuel Koch, Jörg Stetefeld

**Affiliations:** 1 Department of Chemistry, University of Manitoba, Winnipeg, Manitoba Canada; 2 Department of Molecular Biophysics and Biochemistry, Yale University, New Haven, CT USA; 3 Biotech Research and Innovation Centre, University of Copenhagen, Copenhagen, Denmark; 4 Institute for Dental Research and Oral Musculoskeletal Biology, Center for Biochemistry, Medical Faculty, University of Cologne, Cologne, Germany; 5 Institute for Dental Research and Oral Musculoskeletal Biology, Center for Biochemistry, Center for Molecular Medicine, Medical Faculty, University of Cologne, Cologne, Germany

**Keywords:** Netrin-1, Bioreactor, Sleeping beauty, Extracellular matrix protein production, Biophysical quality control

## Abstract

**Abstract:**

The production of recombinant proteins for functional and biophysical studies, especially in the field of structural determination, still represents a challenge as high quality and quantities are needed to adequately perform experiments. This is in part solved by optimizing protein constructs and expression conditions to maximize the yields in regular flask expression systems. Still, work flow and effort can be substantial with no guarantee to obtain improvements. This study presents a combination of workflows that can be used to dramatically increase protein production and improve processing results, specifically for the extracellular matrix protein Netrin-1. This proteoglycan is an axon guidance cue which interacts with various receptors to initiate downstream signaling cascades affecting cell differentiation, proliferation, metabolism, and survival. We were able to produce large glycoprotein quantities in mammalian cells, which were engineered for protein overexpression and secretion into the media using the controlled environment provided by a hollow fiber bioreactor. Close monitoring of the internal bioreactor conditions allowed for stable production over an extended period of time. In addition to this, Netrin-1 concentrations were monitored in expression media through biolayer interferometry which allowed us to increase Netrin-1 media concentrations tenfold over our current flask systems while preserving excellent protein quality and in solution behavior. Our particular combination of genetic engineering, cell culture system, protein purification, and biophysical characterization permitted us to establish an efficient and continuous production of high-quality protein suitable for structural biology studies that can be translated to various biological systems.

**Key points:**

• *Hollow fiber bioreactor produces substantial yields of homogenous Netrin-1*

• *Biolayer interferometry allows target protein quantitation in expression media*

• *High production yields in the bioreactor do not impair Netrin-1 proteoglycan quality*

**Graphical abstract:**

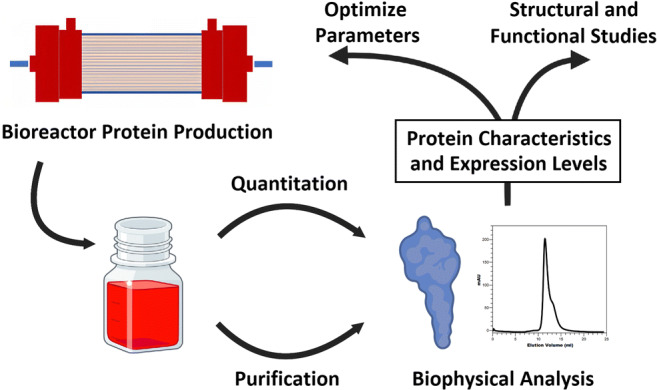

**Supplementary Information:**

The online version contains supplementary material available at 10.1007/s00253-021-11438-0.

## Introduction

Studying the structures and functional behavior of extracellular matrix (ECM) proteins requires large quantities of proteins. Specifically, studying biophysical behavior and determining protein structures can require milligram amounts if a system is to be fully characterized. One such example is the ECM proteoglycan Netrin-1; Netrin-1 is an axon guidance cue, well known for its various signaling functions in cell proliferation, migration, and survival through transmembrane receptors (Boyer and Gupton 2018). These protein-protein interactions can initiate intracellular signaling cascades causing attraction, repulsion, and/or apoptosis (Arakawa 2004) which has significant applications in physiological diseases such as cancer (Mehlen and Furne 2005). The protein structure is comprised of an N-terminal laminin-like domain VI, followed by three epidermal growth factor domains (V-1, V-2, and V-3) (Grandin et al. 2016) and a positively charged C-terminal domain able to interact with heparan sulfate proteoglycans (Kappler et al. 2000). Although the Netrin family of proteins has been studied for some time, the guidance mechanisms are still not fully understood which would allow for the design of effective treatment for various physiological disorders (Arakawa 2004; Méneret et al. 2017). In addition to this, production of Netrin-1 poses some difficulties as glycosylation patterns vary among collections which affects the folding, solubility, and potential binding to receptors.

The first step in studying the molecular structure and function of proteins such as Netrin-1 is to produce the protein at high quality and quantities. This can be challenging as protein expression is highly variable and can yield inconsistent quantities. Our current system involves growing a pool of stably transfected cells in static culture followed by laborious processing of 500 mL spent media harvested every second day. After the cells have reached confluency and are induced for expression, the yield can reach maxima of up to 15 mg/L of Netrin-1 glycoprotein. Although this optimized yield was a considerable increase compared from the initial 1 mg/L, further yield increases were still desirable. Thus, we started to explore more efficient ways in protein production, while maintaining the protein quality of classical culture flask methods.

Here, a hollow fiber bioreactor (HFBR) presented an opportunity to set up a continuous protein production system, with the advantage of harvesting smaller volumes containing higher concentrations of protein. HFBR bring a versatile tool to the research laboratory for continuous production of viruses (Tapia et al. 2016) and recombinant proteins alike (Menshutina et al. 2020). The HFBR system is a capillary bed unit, designed and developed in the early seventies based on the flow of the mammalian circulatory system (Knazek et al. 1972; Knight 1989). This system consists of a cartridge unit packed with hollow fibers where cells grow and proliferate under mostly closed physiological conditions. In the HFBR, culture media flow through a cartridge with two compartments: the intra-capillary space (ICS) and the extra-capillary space (ECS). The ICS is the internal lumen of the capillaries through which the media travels. During the perfusion of media through the ICS, the semi-permeable capillary wall membrane, with a molecular weight cut-off ranging between 5 and 20 kDa, facilitates a constant exchange of nutrients and waste products with the ECS. The ECS is the space between parallel fibers where cells can grow adherent to the capillary walls (Watson et al. 2016) or freely in suspension surrounding the external face wall of each capillary. Nutrients and oxygen continuously move into the ECS while metabolic waste products are constantly removed by the perfusion action of media which is recirculated through the system with a low risk of mechanical damage as would happen with the blades of a normal bioreactor (Menshutina et al. 2020). These semi-permeable capillary membranes provide a larger surface area for a smaller volume as compared to stationary growth flasks. Cells inside the ECS can reach very high cell density (upwards of 10^9^ cells) and secreted protein can easily be collected from one of the ECS outlet ports at higher concentrations in comparison to other systems. The cell density achieved in the HFBR surpasses that of traditional tissue culture flasks, roller bottles, or HYPERFlask® vessels, resulting in higher levels of secreted protein per volume collected (Kearney et al. 2017). The HFBR provides a compact, scalable, and efficient vessel for cells to grow in high density: an artificial system which is designed to mimic how cells grow in vivo.

Although high protein quantities are necessary, protein quality provides the basis for useful experimental data that is collected from functional and structural studies. To assure that both criteria are met, final purified protein should be examined closely. For this, biophysical techniques including dynamic light scattering (DLS) and size exclusion chromatography – multi angle light scattering (SEC-MALS) can provide appropriate data that examines the homogeneity. Furthermore, these methods analyze the shape and size of macromolecules which can function as quality control measurements of produced proteins (Stetefeld et al. 2016).

Combining these techniques with a HFBR in addition to a more efficient transfection system and a streamlined detection system allowed us to continually produce high levels of homogenous Netrin-1 at excellent quality over a span of 5 months. This study did not only provide us with an effective research tool for Netrin-1, but also serves as a novel integrative production model that can easily be translated to the expression of other ECM proteins.

## Materials and methods

### Modification of the vector and cloning of Netrin-1

**Fig. 1:**
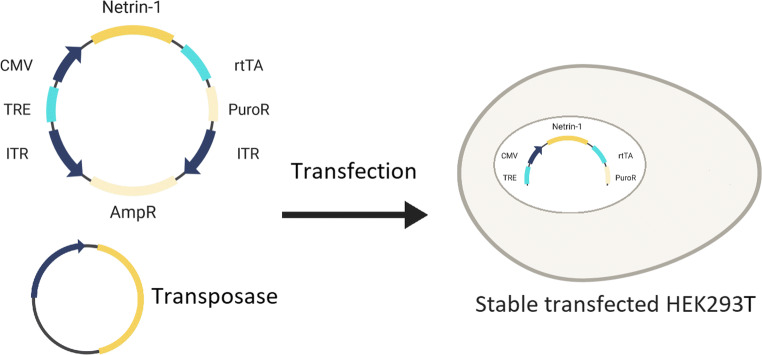
Representation of the GOI inserted into the sleeping beauty transposon system. Both, the sleeping beauty vector containing the GOI and the plasmid bearing the transposase are used to transfect HEK293T cells. The terminal-inverted sequence ITR carries the recognition sequences for the transposase, flanking the cargo in the inverted orientation. Antibiotic resistance genes are included in the vector for future cell selection processes. After chromosomal integration of the GOI, the cell is able to produce Netrin-1 protein in the presence of the inducer doxycycline

Netrin-1 from *Gallus gallus* (NP_990750, aa: 26 – 458) lacking the C-terminus domain was codon optimized for mammalian expression (MW651984), synthesized (Thermo Fisher Scientific), and cloned via the two restriction sites *Nhe*I/*Bam*HI into the generated vector (Fig. 1). We refer to this construct as Netrin-1ΔC. To adapt the sleeping beauty vector for our expression and purification system (gift from Rolf Marschalek (Wächter et al. 2014)), several modifications were included. First, we removed the green fluorescent protein (GFP) gene from the vector since the transfection rate with FuGENE® HD is very high and visualization of successful transfection was unnecessary. Secondly, we added the sequence for the Tet-response element (TRE), the BM-40 signal peptide (MRAWIFFLLCLAGRALAAPLE), a multiple cloning site (MCS), followed by the thrombin cleavage sequence (LVPRGS) right before a double Strep-tag II sequence (SWSHPQFEKGGGSGGGSGGGSWSHPQFEKSG). The double Strep-tag II was necessary to confer strong adhesion to the Strep-Tactin matrix during downstream purification steps. Finally, the sequences for the enzymes *Nhe*I, *Xho*I, *Not*I, and *Bam*HI were introduced into the MCS. The *Bam*HI site in the original vector backbone was mutated and two DNA insertion sites were added adjacent to the two transposase recognition sites.

### Transfection and clonal selection

HEK293T cells, cultured in DMEM/F12 and 7% fetal bovine serum (FBS), were co-transfected with 7 μL Fugene HD (Promega) in a six-well plate with 1.8 μg of the Netrin-1ΔC containing vector S100X and 0.2 μg of the transposase coding plasmid. One day after the transfection, media were changed and supplemented with 3 μg/mL puromycin for tight selection of cells containing the gene of interest (GOI). After 4 days, the cell pool was further propagated in T-75 flasks with DMEM containing 10% (FBS). Upon reaching confluency, it was stored in liquid nitrogen for further use. All cells were cultured at 37 °C in an incubator with humidified atmosphere and 5% CO2.

Clonal selection of the cell pool was done to choose a high-producing clone and to reduce the heterogeneity of the glycans. Cells from a T75 flask were removed with TrypLE (Gibco), and centrifuged at 200 rpm for 3 min. The cell pellet was then resuspended in DMEM containing 10% FBS. Cell count and viability were assessed in a Countess II-FL (Thermo Fisher Scientific) using the trypan blue exclusion method.

The clonal selection was initiated using 96-well culture plates (Corning Costar®) to select those clones that had high resistance to puromycin and were capable of growing faster and expressing high levels of protein. Each well was inoculated with one cell and allowed to adhere and grow until covered more than 50% of the bottom of the well (5–10 days). To select for higher protein production clones, culture media were replaced in each well by DMEM containing 2.5% FBS and 1 μg/mL doxycycline. After 24 h growth, supernatant in each well were transferred to a nitrocellulose membrane (GE Healthcare Life Sciences) in a Dot-Blot apparatus (BIO-RAD). Nitrocellulose membrane was transferred to an iBind™ Western-blot system (Thermo Fisher Scientific) and Strep-tagged protein was detected using Strep-tag® II specific monoclonal antibody conjugated to horseradish peroxidase (IBA). Selected clones were individually stored in cryoprotecting media (FBS with 10% DMSO) in liquid nitrogen.

### Protein expression in a hollow fiber bioreactor

The hollow fiber bioreactor (HFBR) cartridge C2008 with a 5 kDa MW cut-off (FiberCell Systems Inc.) was employed to produce Netrin-1ΔC. A clonally selected HEK293T clone was thawed quickly in a 37 °C water bath and drop wise added to 10 mL pre-warmed DMEM. The cells were removed from the DMSO cryoprotectant by centrifugation at 200*g* for 3 min and the cell pellet was resuspended in fresh DMEM with 10% FBS. The cells were then transferred to a T-75 flask (Corning). Cells grew until 90% cell confluency was reached. Cells were then detached using trypsin-EDTA (TryplE, Gibco) and resuspended in 10 mL of DMEM. Cell viability was measured in a Countess II FL automated cell counter (Thermo Fisher Scientific). Four T-75 flasks were inoculated with 1×10^6^ cells each until 90% cell confluency was reached. Afterwards, the cells were detached, collected, and a total of 10^7^ cells (>98 % cell viability) were resuspended in 20 mL of DMEM and seeded into the ECS pre-equilibrated with 250 mL of DMEM containing 10% FBS (FiberCell Systems). The HFBR unit was placed inside a 37°C incubator with a humidified atmosphere containing 5% CO_2_. DMEM with 10% FBS (Gibco) was recirculated from the reservoir bottle into the cartridge using a proprietary positive pressure displacement pump (FiberCell Systems Duet Pump). When cells in the ECS had consumed 50% of the initial glucose concentration, the volume of media in the reservoir bottle was increased to 500 mL by an addition of 250 mL DMEM with 10% CDM-HD. Upon continued glucose depletion, the media volume was further increased to 1 L by an addition of 500 mL DMEM with 10% CDM-HD. This method is done to initially propagate cells in the presence of FBS and then gradually adapt them to the serum CDM-HD (FiberCell Systems) for more efficient protein production. CDM-HD is a chemically defined serum designed to replace FBS which supports high-density cell cultures. This 1 L reservoir is replaced with fresh DMEM containing 10% CDM-HD upon a >2 mg/mL glucose cell consumption. To start protein expression, doxycycline was added to the culture media in the reservoir bottle. Doxycycline concentrations varied from 0.0625 to 2.0 μg/mL and were adjusted to 1.0 μg/mL for optimal production. During protein expression, a volume of 20 mL was collected from one of the ECS outlet ports with a sterile syringe, clarified by centrifugation at 22,000*g* for 50 min and stored at −20 °C until called upon for downstream processing or analysis.

### Glucose and L-lactate measurements

To measure the carbohydrate level in cell growth media, an aliquot of 1 mL was taken daily from the reservoir bottle and the ECS collections. Glucose and lactate were measured with an automatic glucose analyzer YSI 2700 SELECT^TM^ biochemistry analyzer, (YSI Inc.) following the manufacturer’s protocol.

### Netrin-1 quantitation

Netrin-1 quantitation was performed using a ForteBio Octet K2 system (Sartorius) based on bio-layer interferometry (Do et al. 2008). Specifically, an anti-Netrin1-Fab fragment was biotinylated using amine reactive biotin EZ-Link™ NHS-Biotin (Thermo Fisher Scientific). The biotinylated anti-Netrin1-Fab was immobilized on the ForteBio Streptavidin (SA) Biosensors and equilibrated in collection media (DMEM, 5% FBS, 1 μg/mL doxycycline). Next, a standard curve ranging from 1 to 100 μg/mL of Netrin-1ΔC in collection media was established by measuring the initial binding rate over the first 5 s of association. The binding rate was then plotted against the known concentration to achieve a standard curve (Table S1). Original HFBR samples were diluted in collection media by a factor of 3 to allow for signal measurements within the given standard curve range. Binding rates of all samples were measured, and protein quantities were then calculated. All measurements were performed for 80 s at 25 °C and 1000 rpm shake speed using the initial rate over 5 s for analysis. Data were processed with the ForteBio Octet Data Analysis HT software.

### Protein purification

Netrin-1ΔC was purified by affinity chromatography using a 5 mL Strep-tactin Superflow Plus cartridge (Qiagen) equilibrated with 50 mM Tris, pH 8, and 500 mM NaCl. Each ECS collection was loaded onto the column and washed with 3 column volumes of 50 mM Tris, pH 8, 500 mM NaCl followed by 5 column volumes of 50 mM Tris, pH 8, and 1 M NaCl to eliminate non-specific binding. Protein elution was performed with 2.5 mM of d-Desthiobiotin (MilliporeSigma) in 50 mM Tris, pH 8, and 0.5 M NaCl. The Strep-tag was removed with 1 unit of Thrombin (MilliporeSigma) per mg of protein in a dialysis bag with 25 kDa molecular weight cut-off (Repligen, Spectra/Por) in 50 mM Tris, pH 8, 1 M NaCl, 0.15 M glycine, and 2.5 mM CaCl_2_ and incubated overnight at room temperature. Finally, thrombin was removed using a Benzamidine column (HiTrap® Benzamidine FF, Cytiva) following the manufacturer’s protocol. Tag-free protein was analyzed and purified using an ÄKTA FPLC system with a Superdex 200 10/300 GL column (Cytiva, Healthcare), pre-equilibrated with 50 mM Tris, pH 7.5, and 1 M NaCl. Purified Netrin-1ΔC was concentrated to 1 mg/mL (using molecular weight of 49.5 kDa and extinction coefficient of 49455 M^−1^ cm^−1^ obtained from ProtParam (ExPASy Server)) and stored at 4 °C until further use.

### Analysis of purified protein quality

Proteins were separated on a sodium dodecyl sulfate polyacrylamide 8% gel (SDS-PAGE). Total protein concentration of each supernatant sample was determined using the Bradford method and 20 μg of protein was loaded into each well. An 8% SDS-PAGE gel was run at a constant 10 watts in a PowerPacTM HC (BIO-RAD) using SDS–PAGE running buffer (25 mM Tris base, 200 mM glycine, 0.1% w/v SDS). The gel was stained with Coomassie G250 to visualize the protein bands.

Purified Netrin-1 was analyzed for particle size distribution and hydrodynamic radius by DLS a Zetasizer Nano S (Malvern Instruments). The protein solution was dialyzed into 50 mM Tris, pH 7.5, and 200 mM NaCl and concentrated to varying concentrations ranging from 0.5 to 9.0 mg/mL for measurements. Measurements were done in triplicates at 20°C.

Furthermore, SEC-MALS measurements of Netrin-1 were performed. For this, protein was concentrated to 5 mg/mL in 50 mM Tris, pH 7.5, and 200 mM NaCl and 300 μL samples were run on a Superose 6 Increase 10/300 (Cytiva) column at 0.3 mL/min. MALS measurements were taken using a Wyatt Dawn Heleos II (Wyatt Technology). For calibration and analysis, bovine serum albumin (BSA) was concentrated to 6 mg/mL and also measured on the same system.

## Results

### Genome integration of Netrin-1 using the sleeping beauty transposon system

In initial studies, the transient expression of the full-length (FL) Netrin-1 was relatively low and purification was difficult to achieve due to instability of the protein in solution. Previous transient Netrin-1 expression in human embryo kidney (HEK) 293T cells using the episomal pCEP vector was not suitable for our HFBR system due to its low expression of Netrin-1. For 1L of DMEM, only around 1 mg of purified Netrin-1 was obtained after several steps of protein purification. To circumvent the low expression, we decided to first optimize the codon sequence of Netrin-1 for mammalian expression and to remove the C-terminal domain. Secondly, we subcloned the cells after selection with puromycin. In all further studies, this construct of Netrin-1 missing the C-domain was used.

Due to low expression of Netrin-1 from previous transient transfections, we decided to use an alternative stable transfection method based on the sleeping beauty transposon system, a transposase-mediated DNA genome integration technique (Izsvák et al. 2009). PiggyBac and sleeping beauty are the two commonly used systems for transposon genome insertion which provide enhanced genome integration compared to other stable transfection methods (Izsvák et al. 2009; Balasubramanian et al. 2016). The sleeping beauty system requires two components, the sleeping beauty vector bearing the GOI and a plasmid encoding the transposase information (Fig. 1) for gene integration (Wächter et al. 2014). During transfection, the transposase protein binds to two recognition sites of approximately 200 bp in length on the pITR-TTP sleeping beauty vector and excises the GOI. The transposase then takes the excised GOI and integrates it randomly in between two TA sequences in the cell genome multiple times (up to 20 integration events per cell have been observed). Due to this high gene integration rate, much higher concentrations of puromycin (3 μg/mL) can be used for a stringent selection of clones with increased Netrin-1 expression. In our experience, our sleeping beauty transposon system strategy is comparable to transfection with lentivirus (90%) (Elegheert et al. 2018), but with a quicker selection of stably transfected cells and without the trouble regarding increased biosafety level documentation and regulations requirements. Furthermore, our strategy does not have a gene insertion size limitation, rendering it more generally applicable for larger inserts.

### Selection of the highest producing clone

**Fig. 2:**
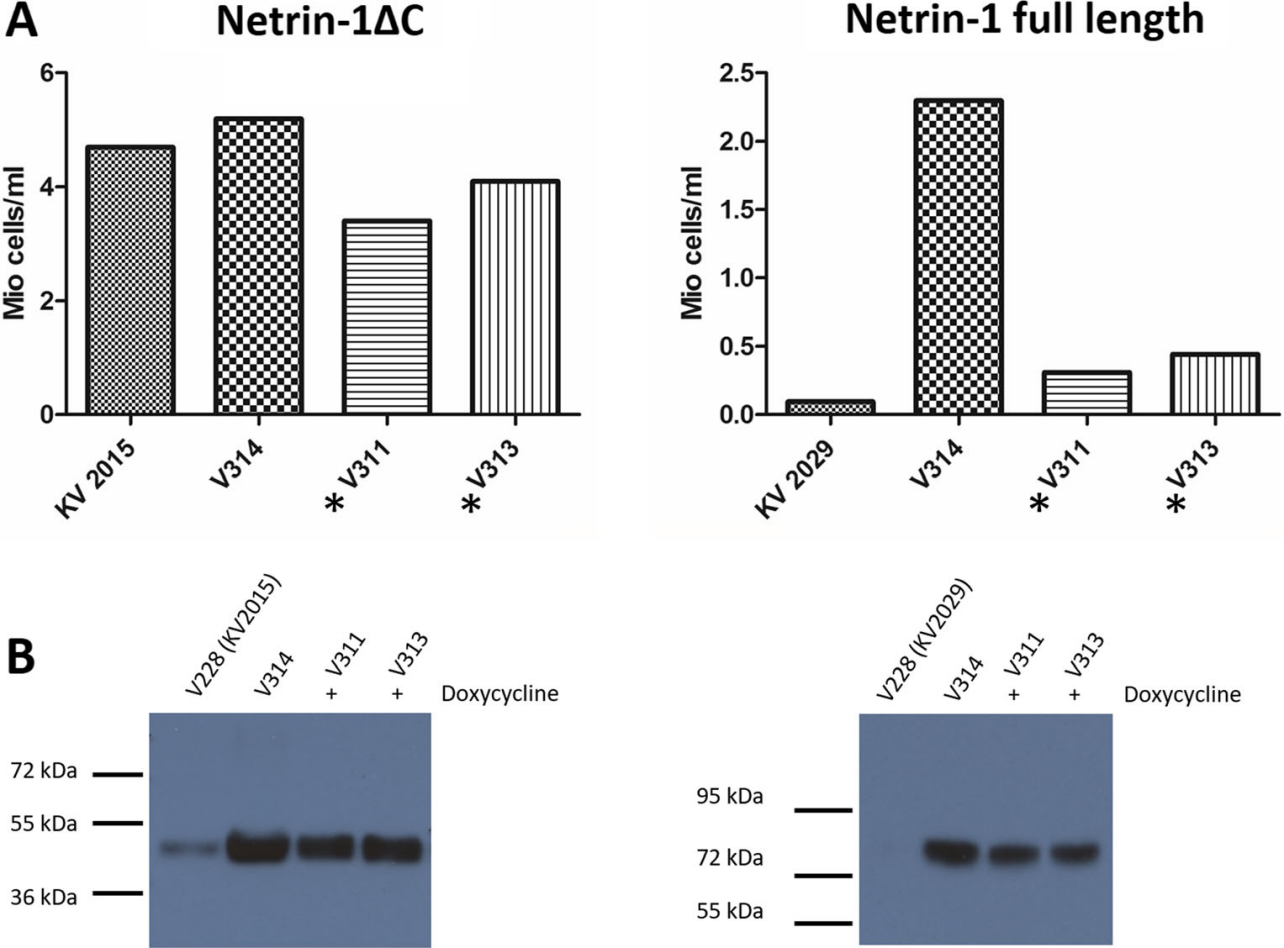
Cell growth and protein expression profiles for Netrin-1 lacking the C-terminal domain (Netrin-1 ΔC) and full length Netrin-1. Profiles are compared between initial strains (KV2015/KV2029, episomal vector system) and varying clones after clonal selections (V311 inducible including GFP/V313 inducible/V314 CMV non-inducible). **A** Cell counts for clones transfected with Netrin-1 constructs and selected for using antibiotic resistance. Netrin-1ΔC constructs have higher cell counts, indicating better vector integration and stability. **B** Each clone produces the desired Netrin-1 protein after selection and purification

To increase protein expression standards, we selected the highest producing clone using two rounds of clonal selection: first for higher gene integration and second for higher protein secretion. Selection for high gene integration was accomplished using high concentrations of puromycin to select for the most resistant clone containing more copies of the GOI in the chromosome. Selection for the high protein secretion clone, we took advantage of the Tet-On inducible system inserted that controlled the expression of GOI and a dot-blot system to select for high protein expression. In our design, we engineered the Netrin-1 gene to be under the control of the Tet-On doxycycline inducible system (Urlinger et al. 2000; Das et al. 2016a; Das et al. 2016b). A gene under the control of a Tet-On system is activated only in the presence of doxycycline that makes the reverse tTA (rtTA) to bind the promoter *P*_*Tigh*_ (a modified Tet-Responsive Element (TRE_mod_)) and switch-on the expression of the regulated gene (Fig. 1). The Tet-On inducible system also permitted us to control the recombinant expression of Netrin-1 during the protein production process. The clone that was chosen for the HFBR was V311 due to high gene integration, stability and protein production (Fig. 2).

### Expression of Netrin-1 in a HFBR

**Fig. 3:**
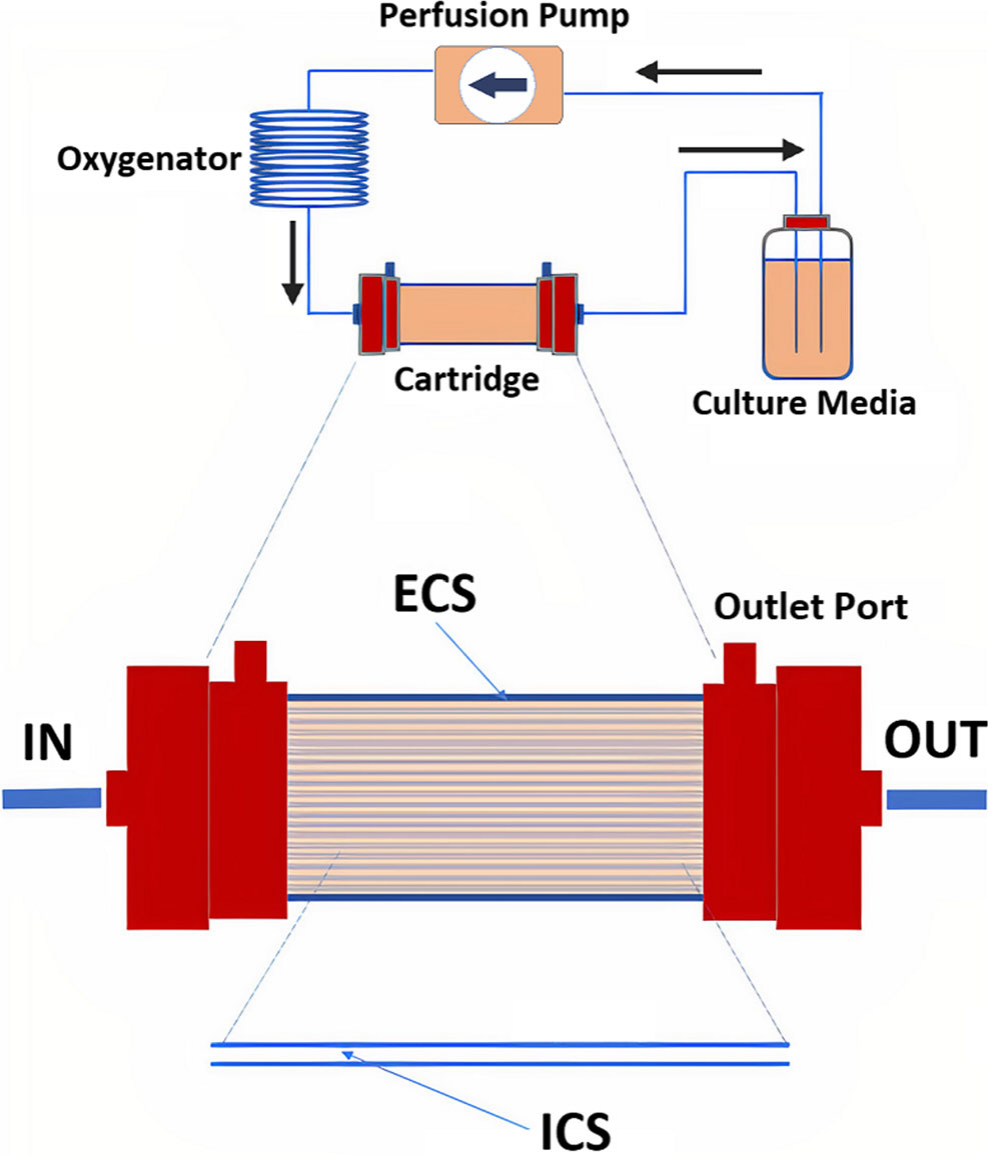
Diagram of a hollow fiber bioreactor. The set-up consists of a reservoir bottle with the culture media, a proprietary positive pressure displacement pump, oxygenator, and a cartridge. The cartridge contains two compartments, the intra-capillary space (ICS) where the media flows through the fiber interior and the extra-capillary space (ECS) where the cells secrete proteins. Capillary walls are permeable to small molecules (typically 10–30 kDa molecular weight cut-off) and allow a continuous exchange of media and waste. Gas exchange takes place while the medium is flowing through the system. The cartridge contains an outlet port to the ECS for expression media extraction

We initially took our high-producing clone, V311, and expressed Netrin-1 in the HYPERFlask® multilayer vessels. Our yields had already increased substantially (from 1 to 15 mg/L) but our throughput was slow and interrupted. For instance, once cells are confluent in the HYPERFlask®, collection of spent media containing Netrin-1 is limited. This is due to the loss of cell viability over time, resulting in reduced protein expression yields for each collection. The HFBR circumvents this with a constant protein yield for each collection with presumably unlimited collections. This is possible because the HFBR is kept in a continuous flow of media and daily removal of protein and dead cells (Fig. 3). The throughput is also increased with only 20 mL to process downstream (instead of >500 mL) to achieve the same protein quantity as in the HYPERFlask (Kearney et al. 2017). Using the HFBR, we assured a consistent and continuous recombinant protein production. The high-density cell growth reduced serum dependency and proportionally increased protein production in the presence of the inducer doxycycline (Kistner et al. 1996; Loew et al. 2010).

**Fig. 4:**
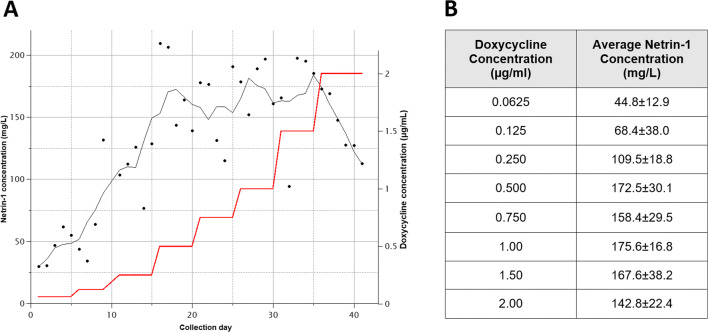
**A** Five point moving average (black line) of Netrin-1 quantities in expression media produced in the hollow fiber bioreactor over 41 days at varying doxycycline concentrations (red line). Starting on day 1 at 0.0625 μg/mL, doxycycline concentrations were gradually increased to a final concentration of 2.00 μg/mL, maintaining each concentration over a 5-day period. **B** Average Netrin-1 concentrations in the extracted media at varying doxycycline concentrations. Protein concentrations were determined using the initial binding rate to a Netrin-1 specific Fab measured by BLI

To optimize growth conditions and protein expression in presence of inducer, we studied the levels of Netrin-1 secreted by the cells at increasing concentrations of doxycycline over time (Fig. 4). Although we determined that the maximum protein concentration was associated with a 1 μg/mL doxycycline concentration (Fig. 4), further increasing it to 2 μg/mL only minimally decreased protein yields. Analysis of expression samples via SDS-PAGE also showed the emergence of the tagged Netrin-1 band as the concentration of doxycycline increased (Fig. S1). Still, internal HFBR doxycycline concentrations were adjusted to 1 μg/mL for continuous protein production.

### Monitoring cell metabolism in the bioreactor

During the experiments, we kept the concentration of glucose and lactic acid at optimal levels. Failures to maintain the correct levels of nutrients in the HFBR will disturb cell growth and induce apoptosis of cells, resulting in poor protein production. In the HFBR, glucose was maintained > 50% of the initial glucose level for optimal growth conditions. When cells had consumed more than 2 mg/mL of glucose, culture media were replaced with fresh media (Fig. S2). These media changes avoided low levels of nutrients, decreased the recirculation of waste products, and maintained a stable production of protein. Accumulation of expression by-products, such as lactate and ammonia inhibit cell growth, protein expression, and may modify protein glycosylation patterns (Yang and Butler 2000; Karengera et al. 2017a; Karengera et al. 2017b). We monitored the glucose and lactic acid levels inside the ECS upon protein collection and exchanged media accordingly to reduce by-product effects for optimal cell metabolism.

In addition, we also monitored the cells harvested during ECS collections using an EVOS FL microscope (Invitrogen) weekly to check cell morphology, growth characteristics, and look for the presence of contamination in the system. No contamination was found in the system throughout the 5 months that the HFBR was in production.

### Protein quantitation in expression media

As part of the Netrin-1 production, we were able to measure protein quantities directly in collection media which greatly assisted us in assessing and analyzing internal HFBR conditions. Protein quantities were determined by combining biolayer interferometry (BLI) (Abdiche et al. 2008) with an antibody fragment (Fab) specifically binding to Netrin-1.

For our purposes, the Netrin-1 specific Fab was biotinylated and immobilized on the BLI biosensors where it would interact with the Netrin-1 present in the media. A standard curve was established relating known concentrations of Netrin-1 (1–100 μg/mL) to the initial binding rate to the Fab (Fig. S3). Following the same method, the protein quantities in the expression media samples were determined (Table S2). By monitoring the protein quantities over time and changing internal conditions, we carefully controlled the expression process (Fig. 4A). Through this, we were able to verify which concentrations of doxycycline were optimal for our system and maximize the Netrin-1 production to 176±17 mg/L at 1 μg/mL of doxycycline (Fig. 4B): approximately four times the amount as compared to 45±13 mg/L at 0.0625 μg/mL doxycycline. Furthermore, knowing the concentrations of Netrin-1 for each collection, we were able to make predictions on expected final protein yields for subsequent experiments. It is worth mentioning that individual measurements were done in a short time frame and high throughput fashion where the initial standard curve was used for all downstream analyses.

### Netrin-1 quality examination

Netrin-1 expression yields were drastically increased using the HFBR. However, high protein production can put strains on cell metabolism which can affect the quality of protein produced (Hotamisligil 2010). For example, high protein production can result in unfolded protein which would precipitate out of solution or partially unfolded protein with altered characteristics (Gregersen and Bross 2010). To ensure we maintained high quality protein using the HFBR, we performed various quality assurance experiments.

**Fig. 5:**
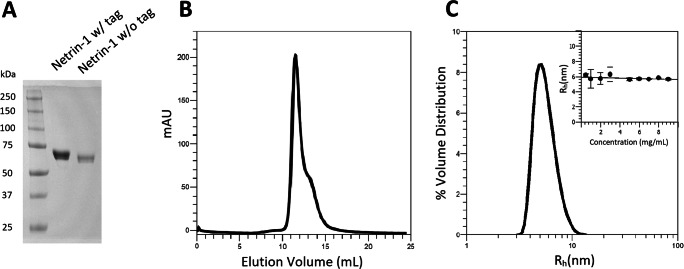
Netrin-1 is produced in high quality. **A** Netrin-1 products before and after cleavage show a clear shift in size corresponding to the loss of the Strep-tag. Both products produce narrow bands with no impurities after affinity-tag purification. **B** Size-exclusion chromatogram of Netrin-1 without a tag. The profile shows the monomer/dimer solution behavior of Netrin-1 and absence of protein aggregation. **C** Particle size distribution by volume of Netrin-1 with a hydrodynamic radius of 5.81 ±0.24 nm which matches the structural data (Krahn et al. 2019). Multiple measurements were taken at increasing concentrations up to 9 mg/mL with no aggregation observed

First, Netrin-1 was purified from the expression media by affinity tag purification and underwent removal of the affinity tag. During these steps, no protein precipitation was observed and the SDS-PAGE gels showed pure Netrin-1 with and without tag (Fig. 5A). The protein bands match previously produced untagged Netrin-1 where a narrow band is observed at around 60 kDa indicating little heterogeneity in the N-glycosylation patterns. These bands also match Netrin-1 produced in a classical flask (Fig. S4). As previously mentioned, the homogeneity of the post-translational modifications is important for structural studies. Here, the narrow protein band suggests minimal differences showing the advantages of the clonal selection process and regular maintenance of normal nutrient levels in the HFBR. Analogous to this, the SEC elution profile of the purified and tag-cleaved protein shows the monomer-dimer peak with minimal aggregation (Fig. 5B). Furthermore, we examined the particle size distribution of Netrin-1 which showed a monodispersed peak with a hydrodynamic radius of 5.81±0.24 nm (Fig. 5C). This matches the size of previously published protein structures (Grandin et al. 2016; Krahn et al. 2019) and, in addition to not observing any aggregation, is indicative of a stable, correctly folded protein. It is worth noting that DLS is not sensitive enough to distinguish the monomer from the dimer of Netrin-1 but rather acts as a fast routine technique that compares values to previously measured data.

Thus, to completely confirm appropriate solution behavior of Netrin-1, we performed SEC-MALS measurements. This method uses light scattering to measure the molecular sizes of species in a sample separated by size-exclusion chromatography. Experimental data confirmed the monomer-dimer equilibrium of Netrin-1 which corresponded to molecular weights of 52.4 kDa and 104.7 kDa, respectively (Fig. S5). Again, minimal aggregation was observed, further confirming high quality protein products. These parameters and the protein band in the SDS-PAGE did not change over the 5 months of production using the HFBR. Collectively, the data shows that the Netrin-1 quality is not affected by the upscaled protein production and can be used for subsequent biophysical and functional receptor interaction studies.

## Discussion

Producing protein amounts in the milligram range for experiments, particular in the structural and biophysical sciences, can be a challenge. There are many parameters which can be optimized without knowing how they will directly affect the protein yield or quality (Karengera et al. 2017a). To address this issue, we present a novel workflow where we used the ECM protein Netrin-1 as a model system. Following this setup, we continuously produced Netrin-1 at high yields and quality over 5 months.

As with traditional methods for increasing protein expression, we optimized the amino acid sequence of Netrin-1. We determined that the full-length Netrin-1 is less stable due to the positively charged C-terminal domain and that final yields are low, most likely due to instability of the vector. Thus, when inserting the DNA sequence into the sleeping beauty vector, we removed the C-terminal domain. Expressing a truncated version of Netrin-1 should not pose problems for functional studies as previous publications have shown that the C-terminal domain is not necessary for receptor binding, such as to deleted in colorectal cancer (DCC) (Xu et al. 2014). Various genes including the Tet-On/Off system and antibiotic resistance which are needed for further selection processes have to be integrated into the genome by the sleeping beauty transposase. This transposase is present in a separate non-integrating vector which mediates the GOI integration into the host genome as a stable gene. Alternatively, the PiggyBac transposon system, a commercial vector system used by the contract manufacturer LONZA for antibody production, can be applied. Downstream clonal selection then allowed for effective selection of Netrin-1-producing cells (Elegheert et al. 2018). In addition, this strategy isolates an individual clone to produce a homogeneous glycosylated recombinant protein. One of the problems that we have faced before in our recombinant protein production was the heterogeneity from batch to batch and inconsistency of recombinant protein solubility and stability. This is most likely caused by metabolic factors like dissolved oxygen, pH, and glucose level which alter protein expression differently in clone variants (Yang and Butler 2000). By preselecting a viable and isolated clone, control of the cell growth and protein expression was increased. As a result, we not only worked with the higher DNA integrated cells but also with a clone that was able to produce a single species of Netrin-1. This helped to obtain a protein preparation with more homogeneous post-translational modification, stability, and quality. The introduction of this step positively impacted further biophysical studies with this molecule.

After successful integration of the vector and clonal selection, cell culture work was migrated to a HFBR for large quantity production of Netrin-1. Main factors that will affect final yields and quality are nutrient availability, oxygenation, and inducer concentrations (Knight 1989; Menshutina et al. 2020). The HFBR effectively mimics in vivo “organ” conditions to maximize protein production. To explore the optimum balance between cell metabolic rates and protein production, we adjusted doxycycline concentrations. It has been reported that doxycycline can alter glycolysis and oxidative phosphorylation pathways in the cell and may affect recombinant protein production (Ahler et al. 2013; Moullan et al. 2015). High levels of doxycycline produce changes in cell metabolism that could lead to overproduction of toxic molecules that may further reduce cell metabolic and proliferation rates (Ahler et al. 2013), which in our case would be observed as a decrease in Netrin-1 expression and increased cellular waste. Inducer concentrations were gradually increased over the first 41 days to a maximum of 2 μg/mL. Daily samples were taken and Netrin-1 concentrations were determined using BLI. These measurements revealed an optimum doxycycline concentration of 1 μg/mL to which was then used for further protein production.

Determining protein quantities in expression media provided us with many advantages for monitoring internal HFBR conditions and downstream protein processing. Although there are other methods such as ELISA and mass spectrometry techniques which are also protein specific, BLI offers the best combination of accuracy and high throughput analysis to determine protein concentrations (Roh et al. 2011). In addition, if an antibody is available for the protein of interest, protein concentrations can be measured directly from collection media. Presumably, tag-specific antibodies such as to a Strep-tag could also be used to monitor protein quantities. For that purpose, we produced a standard curve with known Netrin-1 concentrations in the collection media to account for potential media induced reading effects. In practice, this technique is extremely fast in measuring protein concentrations which allowed us to select the most adequate collection for downstream processing. Furthermore, monitoring protein production allowed us to determine optimal internal bioreactor conditions and carefully fine tune them. Introducing this technique proved to be highly valuable for our analysis of the HFBR, allowing us to increase our yields tenfold over previously used flask methods.

Finally, producing high amounts of protein does not necessarily imply that the final product is of sufficient quality for experimental studies. High yields of protein are often associated with high metabolic stress which might prevent successful folding (Gregersen and Bross 2010). Non-functional proteins display low stability which tends to present itself in fast aggregation upon experimental handling. These proteins might also have different hydrodynamic radii if certain domains are unfolded. To account for this, we fully purified Netrin-1 and performed quality assurance assays including SDS-PAGE, DLS, and SEC-MALS. Resulting measurements showed minimal protein aggregation and a hydrodynamic radius matching previously published data and protein structure models (Grandin et al. 2016). Netrin-1 also demonstrated normal solution behavior of its monomer-dimer equilibrium. For our quality analysis, we looked for biophysical methods that were fast and easy to integrate into the workflow. DLS is an excellent example of this as light scattering can be measured directly and the method is non-destructive. SEC-MALS can provide additional information about the protein such as molecular weights and oligomerization states. Although this method is more involved, it provides crucial quality information about protein samples that should not be overlooked when going for more intricate biophysical studies.

Following this workflow, we were able to generate a suitable plasmid system for the HFBR and screen a wide range of internal HFBR conditions to maximize production. Therefore, within a few weeks, we had an optimized growth protocol for Netrin-1 and were producing 4 mg of protein each day. This is a drastic increase in productivity compared to our previous use of traditional flasks. At this point, it should be mentioned that the protein quality did not change over the extended monthly course of production. This includes no changes in the glycoproteins physical values, such as molecular weights and hydrodynamic radii, measured through our biophysical analysis indicating consistently homogeneous Netrin-1. Bioreactors for high protein production have been explored previously. However, to our knowledge, an integrative approach which uses a host of techniques to assure that the protein quality is high and consistent has not been introduced yet. The presented workflow does just that. Ultimately, careful protein production combined with biophysical analysis can save a lot of time and effort in the long run by guaranteeing optimal starting material for subsequent studies.

Streamlined protein production offers many advantages for industry and research. Combining bioreactors with simple protein quantitation and quality studies provides an excellent starting point for structural and functional studies. Here, expression systems designed to secrete the desired protein like, in this case, the ECM protein Netrin-1 are ideal for using the HFBR. Although initial efforts and entry costs present a formidable barrier, unmatched homogenous protein amounts can be produced continuously over a long period of time in a labor and cost-efficient manner. Adding to this, monitoring of internal bioreactor conditions through biophysical techniques, like BLI and DLS, proved themselves as powerful tools for quality assurance. The methods described in this study provide an excellent research tool if large amounts of protein are required without compromising protein quality.

## Supplementary Information

ESM 1 (PDF 925 kb)
