# Supplementary material for: Homogenous overexpression of the extracellular matrix protein Netrin-1 in a hollow fiber bioreactor
Source: Appl Microbiol Biotechnol. 2021 Aug 3;105(14-15):6047–57. doi: 10.1007/s00253-021-11438-0 (PMC8390410; doi:10.1007/s00253-021-11438-0)
Supplement: Supplementary file 1 — (PDF 925 kb) [file 253_2021_11438_MOESM1_ESM.pdf]

Homogenous overexpression of the extracellular matrix protein Netrin-1 in a Hollow Fiber Bioreactor – Supplemental Information

Aniel Moya[1], Monika Gupta[1], Fabian Heide[1*], Natalie Krahn[2], Scott Legare[1], Denise Nikodemus[3], Thomas Imhof[4], Markus Meier[1], Manuel Koch[4] and Jörg Stetefeld[1*]

[1] *University of Manitoba, Department of Chemistry, Winnipeg, Manitoba, Canada*

[2] *Yale University, Department of Molecular Biophysics and Biochemistry, New Haven, CT, USA*

[3] *Biotech Research and Innovation Centre, University of Copenhagen, Copenhagen, Denmark*

[4] *Institute for Dental Research and Oral Musculoskeletal Biology, Center for Biochemistry, Medical Faculty, University of Cologne, Cologne, Germany*

* Corresponding Authors:

Fabian Heide, +1(204)332-0853, Heidef@myumanitoba.ca

Jörg Stetefeld, +1(204)474-9731, Jorg.Stetefeld@umanitoba.ca

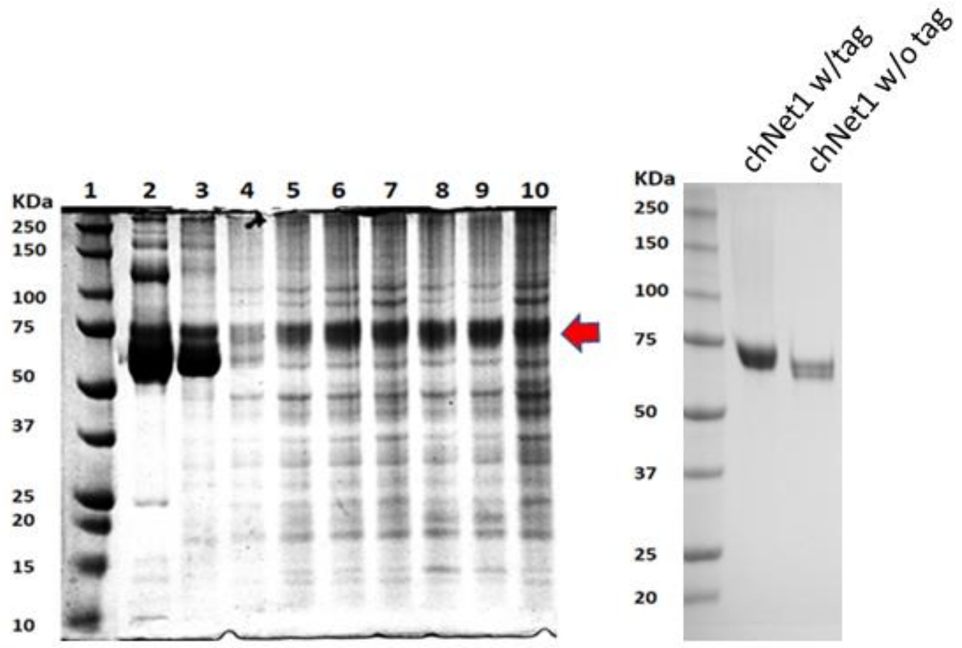

A B

Fig. S1. (A) SDS-PAGE (8%) shows bands corresponding to selected individual ECS collection at different concentrations of doxycycline. Red arrow indicates the region corresponding to the Netrin-1ΔC relative migration band. Lane. (1) Molecular weight ladder, (2) 0 µg/mL Dox, (3) 0.062 µg/mL Dox, (4) 0.125 µg/mL Dox, (5) 0.25 µg/mL Dox, (6) 0.5 µg/mL, (7) 0.75 Dox µg/mL, (8) 1.0 µg/mL Dox, (9) 1.5 µg/mL, (10) 2.0 µg/mL Dox. (B) SDS-PAGE (8%), shows the purified Netrin 1ΔC before and after thrombin cleavage. A clear shift in the molecular weight after thrombin digestion can be observed.

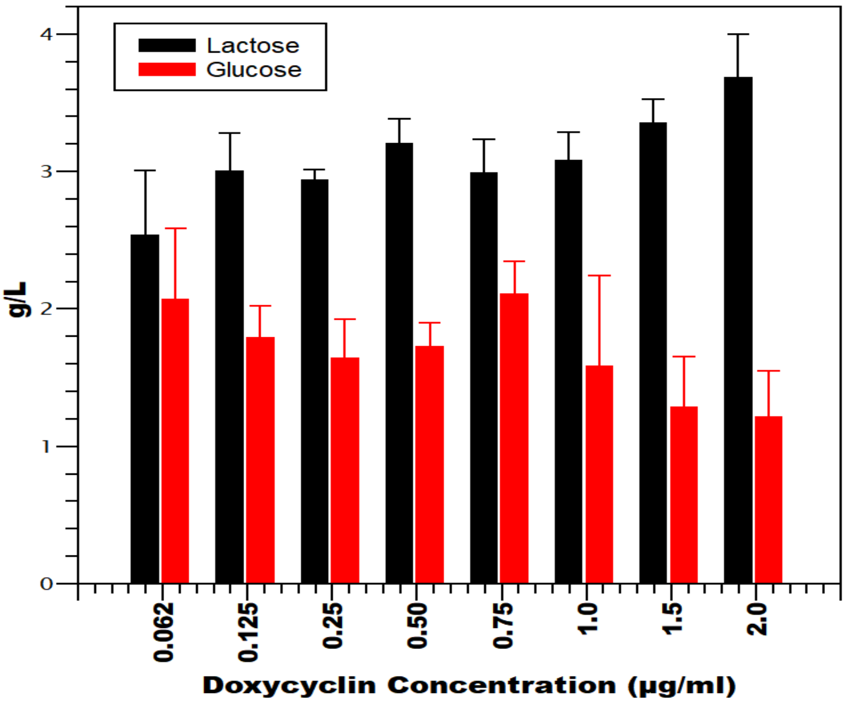

Fig. S2. Levels of glucose (red) and lactic acid (black) in supernatant collected in the ECS at different concentration of doxycycline inducer. Data shown is an average of 3 measurements with error bars corresponding to the standard deviation. A small increase in the level of lactic acid is observed with increasing concentration of doxycycline. The decrease in glucose observed as doxycycline concentration increases indicates boosted cell proliferation and metabolism.

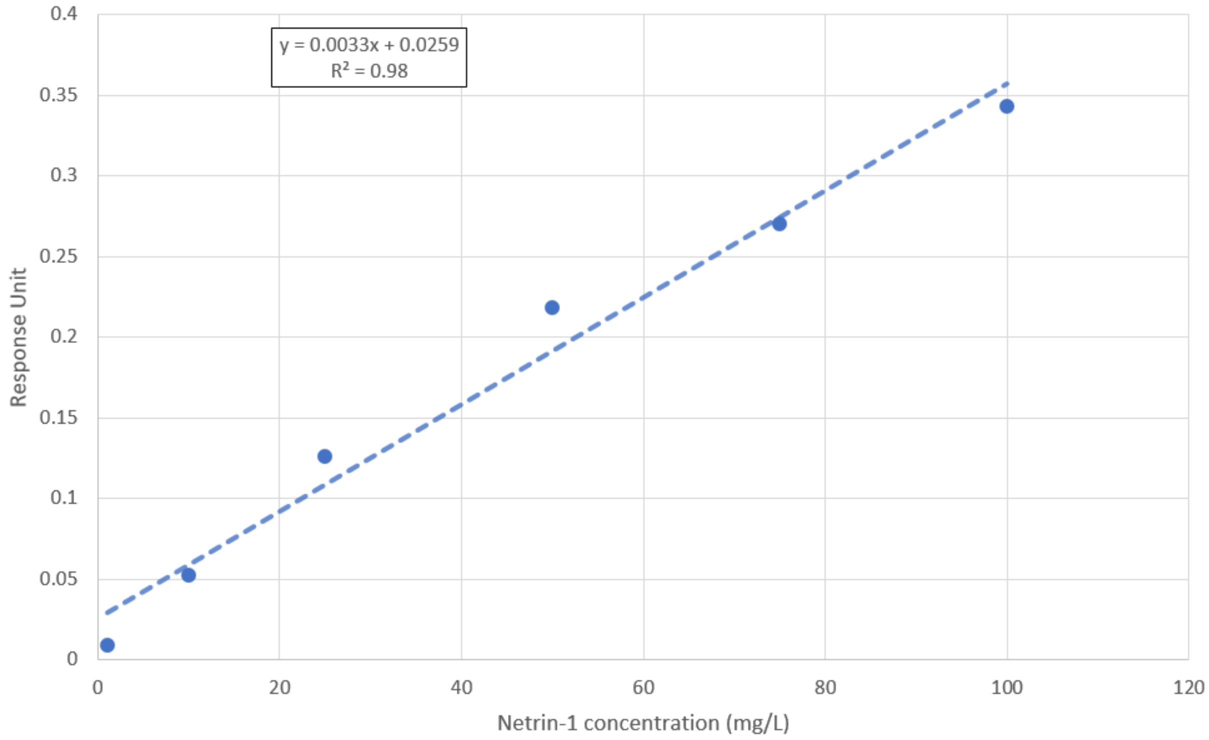

Fig. S3. Standard curve for Netrin-1 quantitation via BLI from which unknown concentrations of Netrin-1 in expression media were calculated. Standards were measured in collection media (DMEM, 5% FBS, 1 µg/mL doxycycline) to account for effects induced by the media. All measurements progressed for 80 seconds at 25°C and 1000 rpm shaker speed using the initial rate over 5 seconds as the response rate for analysis. Data processing was done using the ForteBio Octet Data Analysis HT software. The linear best fit has an R^2 value of 0.98.

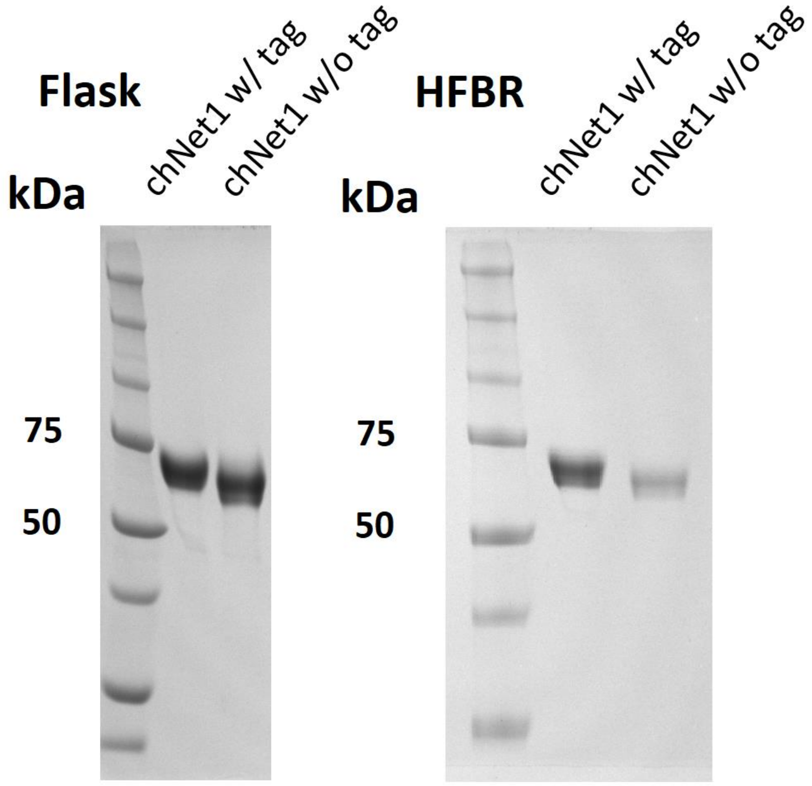

Fig. S4. Netrin-1 final product comparison between different expression methods. Protein migration bands in an SDS-PAGE show that the final products of Netrin-1 (with or without tag) match between the classical flask and the HFBR production methods.

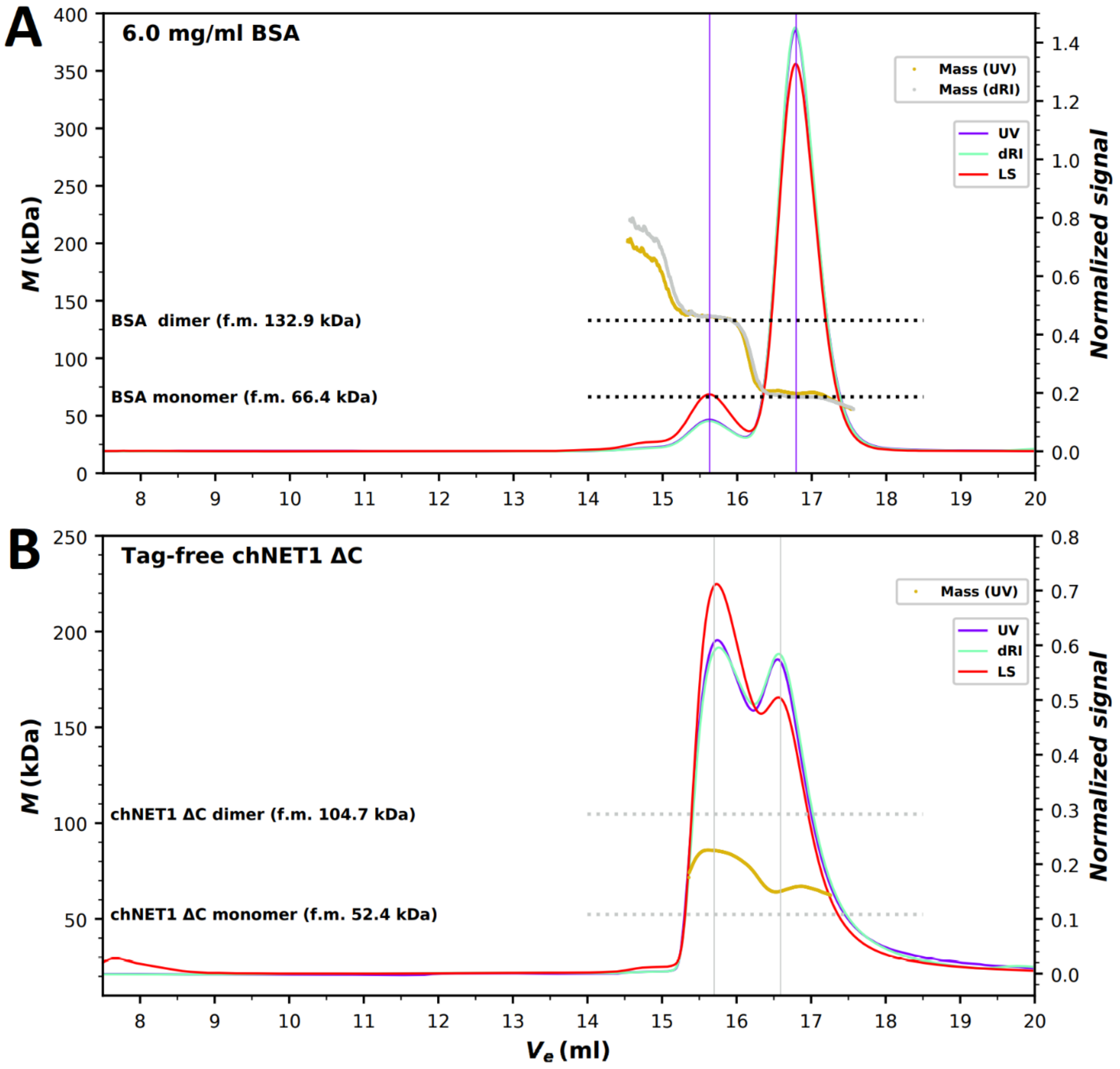

Fig. S5. Purified Netrin-1 quality analysis produced in HFBR using SEC-MALS. (A) Bovine Serum Albumin (BSA), concentrated at 6 mg/mL, was used for calibration and protein size analysis. (B) Light scattering measurements of Netrin-1 were taken at a concentration of 5 mg/mL. Size analysis shows the monomer-dimer equilibrium and the appropriate molecular weights of 52.4 kDa and 104.7 kDa respectively. Minimal aggregation is observed.

Table S1. Calibration values of known Netrin-1 concentrations and their initial binding rate to the biotinylated Fab. All measurements were performed for 80 seconds at 25°C and 1000 rpm shake speed using the initial rate over 5 seconds for analysis.

Netrin-1 w/tag Concentration (mg/L)	Initial Binding Rate (nm/sec)
1.0	0.00885
10.0	0.05291
25.0	0.12643
50.0	0.21818
75.0	0.27065
100.0	0.34332

Table S2. Binding rate values for the samples 1 to 41 and their corresponding Netrin-1 concentrations. All measurements were performed for 80 seconds at 25°C and 1000 rpm shake speed using the initial rate over 5 seconds for analysis. Data processing was done using the ForteBio Octet Data Analysis HT software.

Sample Name	Initial Binding Rate	Dilute Netrin-1 Concentration (mg/L)	Original Netrin-1 Concentration (mg/L)
1	0.05889	9.96	29.89
2	0.05956	10.17	30.50
3	0.07753	15.59	46.76
4	0.09425	20.63	61.90
5	0.08673	18.36	55.09
6	0.07419	14.58	43.74
7	0.06391	11.48	34.44
8	0.09637	21.27	63.81
9	0.17138	43.90	131.70
11	0.14023	34.50	103.51

12	0.15016	37.50	112.49
13	0.16505	41.99	125.97
14	0.11059	25.56	76.68
15	0.16796	42.87	128.60
16	0.25732	69.82	209.47
17	0.2539	68.79	206.38
18	0.18447	47.85	143.54
19	0.20713	54.68	164.05
20	0.17965	46.39	139.18
21	0.22239	59.29	177.86
22	0.22111	58.90	176.70
23	0.17099	43.78	131.34
24	0.15319	38.41	115.24
25	0.23659	63.57	190.71
26	0.2232	59.53	178.59
27	0.19393	50.70	152.11
28	0.23473	63.01	189.03
29	0.24359	65.68	197.05
30	0.20383	53.69	161.06
31	0.20876	55.18	165.53
32	0.13029	31.50	94.51
33	0.24401	65.81	197.43
34	0.24138	65.02	195.05
35	0.23085	61.84	185.52

36	0.21676	57.59	172.77
37	0.21268	56.36	169.07
38	0.18923	49.28	147.85
39	0.16689	42.54	127.63
40	0.16641	42.40	127.20
41	0.15024	37.52	112.57
